# Four laparoscopic surgical strategies for pediatric direct inguinal hernia: a retrospective single-center case series of six male children

**DOI:** 10.3389/fped.2026.1916132

**Published:** 2026-08-25

**Authors:** Yan Xu, Shuaishuai Han, Haixin Ding, Fenghui Zhang, Siyu Liu, Wenxin Jiang, Jizhou Shi

**Affiliations:** 1 Department of Pediatric Surgery, Shengli Oilfield Central Hospital, Dongying, China; 2 Department of Medical Records Management, Shengli Oilfield Central Hospital, Dongying, China; 3 Department of Pediatrics, Shengli Oilfield Central Hospital, Dongying, China; 4 Department of Pediatric Surgery, Dongying District People’s Hospital, Dongying, China

**Keywords:** case series, hernia sac eversion, hernia sac ligation, hesselbach's triangle, laparoscopic hernia repair, pediatric direct inguinal hernia

## Abstract

**Objective:**

Pediatric direct inguinal hernia is a rare subtype of inguinal hernia, characterized by protrusion of abdominal viscera through the weak posterior wall of the inguinal canal without passing through the internal inguinal ring. This condition results from congenital hypoplasia of the transversalis fascia and focal structural defects in the inguinal region, with a lower incidence than pediatric indirect inguinal hernia. Multiple laparoscopic protocols for pediatric direct inguinal hernia have been reported, yet no unified surgical standard has been established, and relevant clinical data lack systematic collation. This retrospective single-center case series summarizes clinical experience of four laparoscopic repair techniques for pediatric direct inguinal hernia, with emphasis on surgical procedures, postoperative complications and long-term hernia recurrence. This study aims to report clinical outcomes of the four laparoscopic approaches and clarify the selection strategy of the four surgical modalities.

**Methods:**

This is a retrospective, single-center, formal consecutive clinical case series. Inclusion criteria: 1) were intraoperatively confirmed as having primary direct inguinal hernia during laparoscopic surgery; 2) had complete electronic medical records, operative notes and outpatient follow-up data; 3) received laparoscopic repair techniques. Exclusion criteria: 1) combined indirect inguinal hernia, femoral hernia or other complicated inguinal region abnormalities; 2) recurrent hernia after prior inguinal surgery; 3) incomplete clinical or follow-up data. All patients underwent laparoscopic direct inguinal hernia repair at Shengli Oilfield Central Hospital between January 2019 and April 2026. Clinical data including age, gender, operation date, surgical technique and complications were retrospectively reviewed and analyzed.

**Results:**

Six male pediatric patients met the inclusion criteria, and none satisfied any exclusion criterion. Among them, five presented with unilateral hernia and one with bilateral hernia. The median age was 3 years and 9 months (range: 2 years and 1 month to 7 years and 3 months). All children were preoperatively suspected to have indirect inguinal hernia by physical examination, while direct inguinal hernia was confirmed intraoperatively. All patients received laparoscopic repair adopting four distinct surgical techniques: laparoscopic eversion and lateral fixation of direct hernia sac, laparoscopic high ligation of direct hernia sac, laparoscopic reinforcement with medial umbilical ligament, and combined laparoscopic high ligation of hernia sac plus medial umbilical ligament reinforcement. All operations were completed successfully with minimal intraoperative blood loss. No intraoperative injuries to the vas deferens, spermatic vessels, testis, bowel, urinary bladder, external iliac vessels or nerves were identified. Oral liquid diet was resumed 6 to 8 h after surgery, and daily activities were restored within 24 h postoperatively. No short-term complications such as surgical site infection or hematoma occurred in any patient. The final follow-up was conducted in June 2026 before manuscript submission, with follow-up durations ranging from 5 months to 82 months. No long-term complications including hernia recurrence, testicular atrophy or chronic inguinal pain were documented in all subjects.

**Conclusion:**

Laparoscopic surgery is safe, reliable and minimally invasive for pediatric direct inguinal hernia. Based on our single-center experience, surgical selection depends on whether the hernia sac is amenable to ligation and whether the medial umbilical ligament can sufficiently cover the Hesselbach's triangle. Large-cohort studies are required to further validate the clinical efficacy of each repair strategy.

## Introduction

Pediatric direct inguinal hernia is a rare type of childhood inguinal hernia. Direct inguinal hernia accounts for only 2%–3% of cases, whereas indirect inguinal hernia accounts for more than 95% (1). Its pathogenesis lies in congenital hypoplasia of the transversalis fascia and local structural defects in the inguinal region. Abdominal viscera protrude outward through the weakened posterior wall of the inguinal canal without passing through the internal inguinal ring, thereby forming a hernia (2). In clinical practice, pediatric direct inguinal hernia is highly likely to be misdiagnosed as indirect inguinal hernia by preoperative physical examination and ultrasonography, and definitive diagnosis can mostly be obtained during laparoscopic exploration. At present, there is no unified and standardized surgical protocol for laparoscopic repair of pediatric direct inguinal hernia, and various surgical options are available clinically. Accordingly, this study summarized four commonly used laparoscopic percutaneous repair techniques for pediatric direct inguinal hernia. Combined with the clinical data of six children, we compared and analyzed the clinical efficacy, safety and application value of different procedures, aiming to provide clinical references and standardize surgical manipulations for laparoscopic treatment of pediatric direct inguinal hernia. This case series has been reported in line with the PROCESS guidelines (3).

## Method

### Inclusion and exclusion criteria

Patients were eligible for inclusion if they: 1) were intraoperatively confirmed as having primary direct inguinal hernia during laparoscopic surgery; 2) had complete electronic medical records, operative notes and outpatient follow-up data; 3) received laparoscopic repair techniques.

Exclusion criteria were: 1) combined indirect inguinal hernia, femoral hernia or other complicated inguinal region abnormalities; 2) recurrent hernia after prior inguinal surgery; 3) incomplete clinical or follow-up data.

### Follow-up protocol

All patients were followed up via outpatient re-examination or telephone follow-up. The last follow-up was conducted in June 2026, with follow-up duration ranging from 5 to 82 months. Outcome data were collected by two independent pediatric surgeons through medical record review and clinical examination.

Six male pediatric patients receiving laparoscopic direct inguinal hernia repair at Shengli Oilfield Central Hospital between January 2019 and April 2026 were retrospectively enrolled in this study. Comprehensive clinical data of all patients were collected and analyzed, including sex, age, preoperative diagnosis, intraoperative confirmed diagnosis, operation date, detailed surgical procedures, operative time, postoperative recovery, complications and long-term follow-up outcomes (Table 1). Four types of surgical procedures were adopted in this cohort, namely laparoscopic eversion and lateral fixation of direct inguinal hernia sac, laparoscopic ligation of direct inguinal hernia sac, laparoscopic medial umbilical ligament reinforcement, and laparoscopic ligation of direct inguinal hernia sac combined with medial umbilical ligament reinforcement. Individualized surgical strategies were selected intraoperatively according to the morphology of hernia sac, size of fascial defect and severity of local tissue weakness of each child.

**Table 1:** Baseline clinical data of six children with laparoscopic direct inguinal hernia repair.

Characteristic	Patient 1	Patient 2	Patient 3	Patient 4	Patient 5	Patient 6
Sex	Male	Male	Male	Male	Male	Male
Age	2 years and 6 months	7 years and 3 months	5 years and 8 months	2 years and 1 month	5 years and 1 month	2 years and 1 month
Preoperative Diagnosis	Indirect Inguinal Hernia	Indirect Inguinal Hernia	Indirect Inguinal Hernia	Indirect Inguinal Hernia	Indirect Inguinal Hernia	Indirect Inguinal Hernia
Hernia laterality	Left	Right	Left	Right	Right	Bilateral
Intraoperative Diagnosis	Direct Inguinal Hernia (Figure 1A)	Direct Inguinal Hernia (Figure 2A)	Direct Inguinal Hernia (Figure 3)	Direct Inguinal Hernia (Figure 4A)	Direct Inguinal Hernia(Figure 5A)	Direct Inguinal Hernia(Figures 6A,B)
Operation Date	2019.08	2022.01	2022.10	2024.03	2024.09	2026.01
Operation Name	Laparoscopic eversion and lateral fixation of hernia sac	Laparoscopic eversion and lateral fixation of hernia sac	Laparoscopic Ligation of Hernia Sac	Laparoscopic Medial Umbilical Ligament Reinforcement	Laparoscopic Ligation of Hernia Sac	Laparoscopic Ligation of Hernia Sac Combined with Medial Umbilical Ligament Reinforcement
Operative time (minutes)	35	6	5	8	6	23
Follow-up duration (months)	82	53	44	27	21	5
Complications	None	None	None	None	None	None

**Figure 1:**
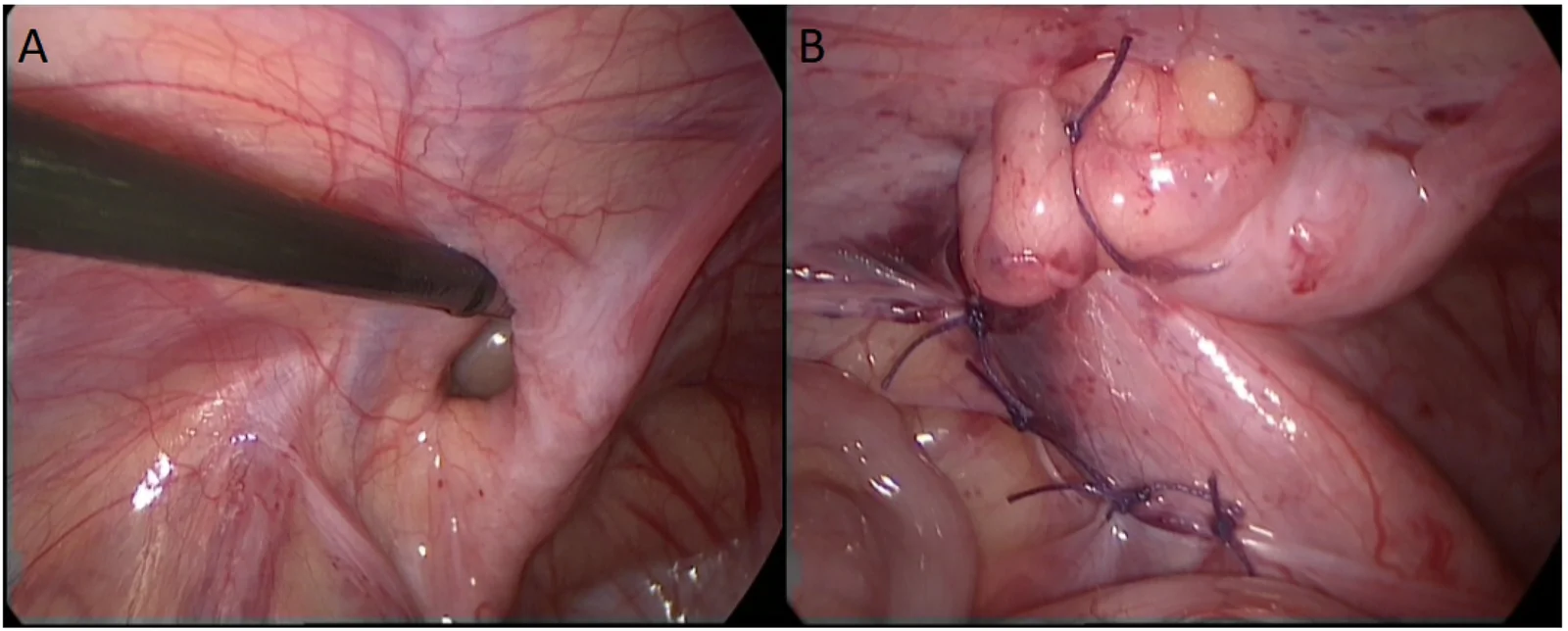
Patient 1. **(A)** Left direct inguinal hernia. **(B)** After repair.

**Figure 2:**
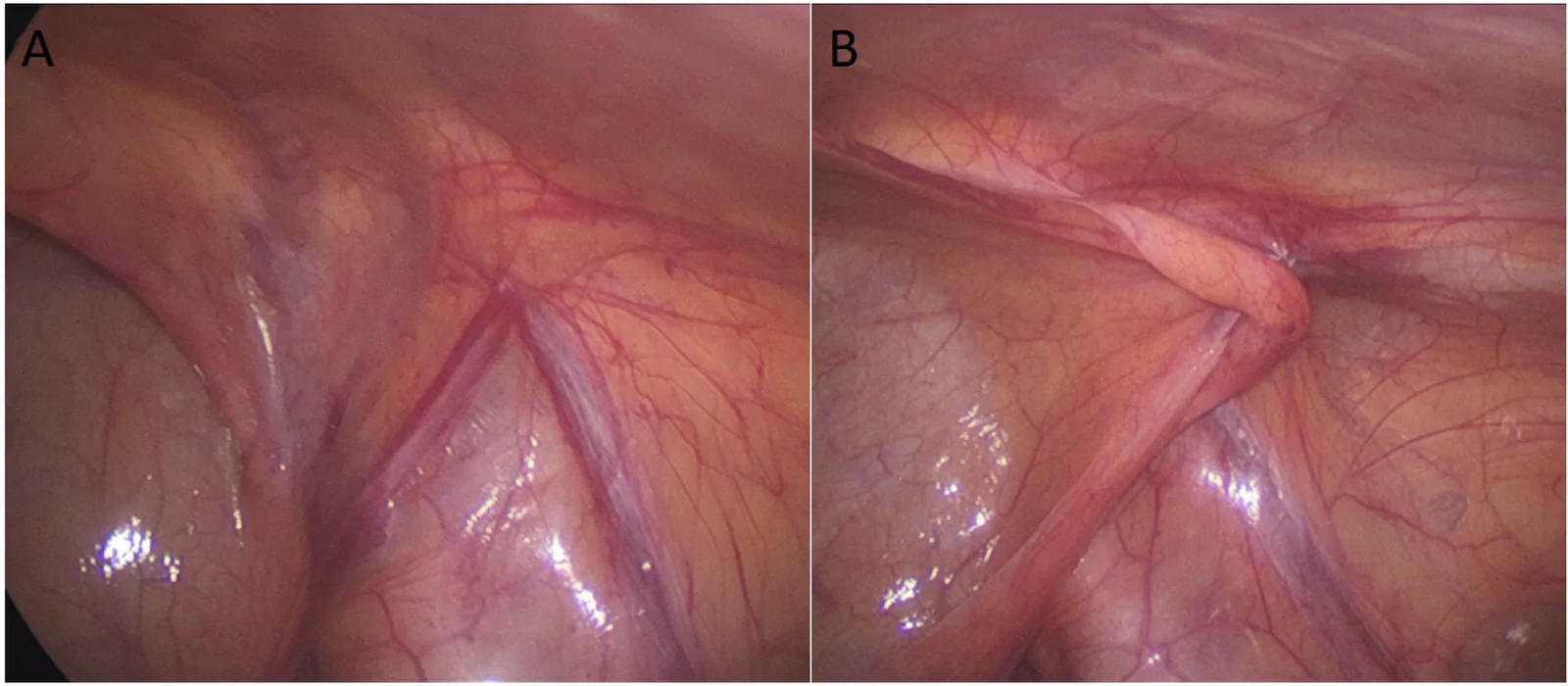
Patient 2. **(A)** Right direct inguinal hernia. **(B)** After repair.

Patient 1 presented with a left direct inguinal hernia (Figure 1A), and Patient 2 had a right direct inguinal hernia (Figure 2A). Both children underwent laparoscopic eversion and lateral fixation of the direct hernia sac. Patient 1 received three 5-mm trocar ports: two were placed at the left and right umbilical margins, and the third was positioned on the left abdomen. For Patient 2, two 5-mm ports were created at the left and right umbilical margins, alongside a 2-mm port for the fascial closure device (spring-loaded two-prong grasper) located in the inguinal region. Surgical procedure of Patient 1: Under direct laparoscopic visualization, the fundus of the direct hernia sac was tractioned to evert the sac. The sac fundus was further pulled lateral to the internal inguinal ring, then sutured and fixed to the abdominal wall. Laparoscopic inspection verified that the everted distal sac was firmly anchored to the abdominal wall and fully covered Hesselbach's triangle (Figure 1B). Surgical procedure of Patient 2: Two 5 mm incisions were made at the left and right umbilical margins for placement of 5-mm trocars (serving as the camera port and working port, respectively). A 2-mm skin incision was created 1–2 cm lateral to the cutaneous projection of the affected internal inguinal ring. A non-absorbable suture was preloaded into the fascial closure device. Under laparoscopic guidance, the fascial closure device percutaneously punctured the abdominal wall and peritoneum vertically to enter the peritoneal cavity. Laparoscopic forceps grasped the fundus of the direct hernia sac and pulled it inward to fully evert the sac. The fascial closure device pierced the hernia fundus and released the suture thread, then withdrew extraperitoneally along the original puncture track, leaving one thread end intraperitoneally and the other outside the abdominal wall. The fascial closure device was reinserted through the same 2 mm skin incision, with its peritoneal entry site several millimeters away from the first puncture point. Its two prongs hooked the pre-placed intraperitoneal suture and pulled the thread back out of the abdominal wall along the track. The suture was tightened extracorporeally. Laparoscopic confirmation was performed to ensure the everted distal hernia sac was securely fixed to the abdominal wall and completely covered Hesselbach's triangle (Figure 2B).

**Figure 3:**
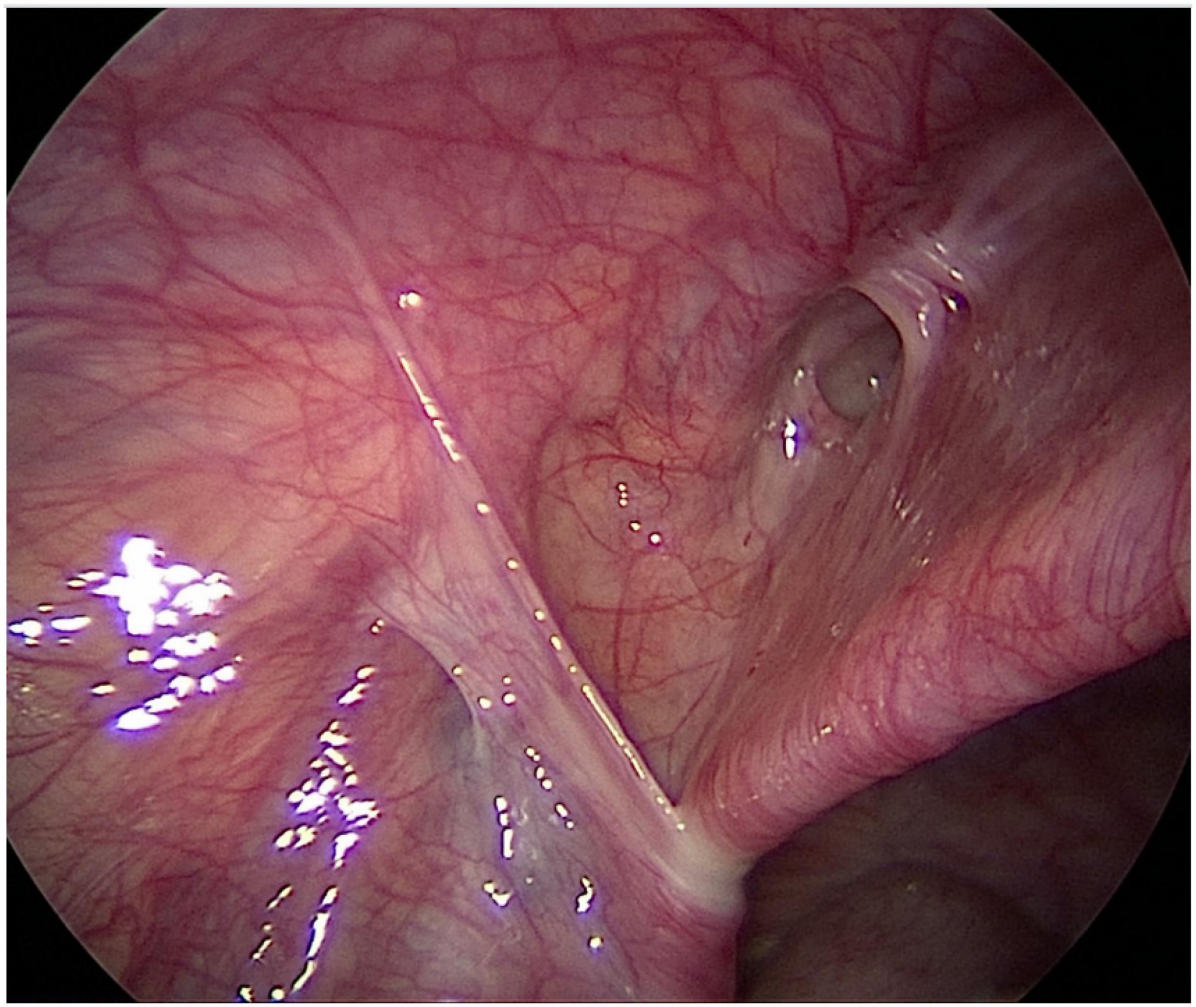
Patient 3. Left direct inguinal hernia.

Patient 3 with left direct inguinal hernia (Figure 3) and Patient 5 with right direct inguinal hernia (Figure 5) underwent laparoscopic ligation of direct inguinal hernia sac. The procedures were performed as follows: Two 5 mm incisions were created at the left and right margins of the umbilicus, into which 5 mm trocars were inserted as the observation port and working port respectively. A 2 mm incision was made at the body surface projection of the hernia sac neck. The fascial closure device was preloaded with 2–0 suture, with one end reserved outside the body and the other clamped between the two jaws. The fascial closure device vertically punctured the abdominal wall, advanced extraperitoneally to the peritoneum medial to the hernia sac neck, continued to travel halfway around the neck, and penetrated the peritoneum beneath the hernia sac neck to enter the abdominal cavity. The handle was pressed to open the jaws and release one end of the suture intraperitoneally, followed by retraction of the fascial closure device extraperitoneally, leaving one suture end intraperitoneal and the other extraperitoneal. The fascial closure device was reinserted through the same incision, advanced extraperitoneally halfway around the lateral side of the hernia sac neck, and entered the abdominal cavity via the same peritoneal puncture site. The two jaws targeted the intraperitoneal suture, opened to grasp the thread upon handle compression, retracted after releasing the handle, and pulled the suture out of the abdominal wall. The suture circumferentially encircled the neck of the direct hernia sac, with both ends outside the body. The suture was tightened extracorporeally. Laparoscopy confirmed complete closure of the internal inguinal ring and high ligation of the hernia sac root. The knot was tied subcutaneously.

**Figure 4:**
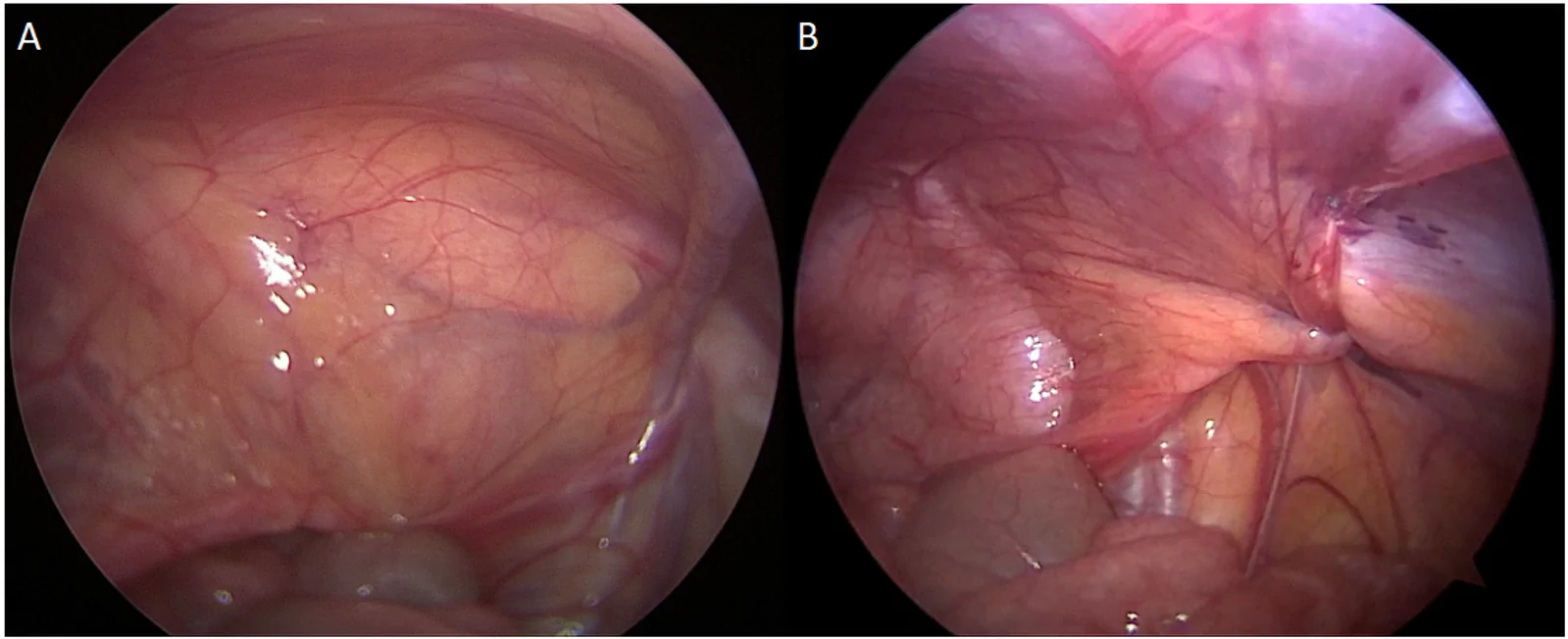
Patient 4. **(A)** Right direct inguinal hernia. **(B)** After repair.

Patient 4 presented with right direct inguinal hernia (Figure 4) and received laparoscopic medial umbilical ligament reinforcement. The reinforcing procedure of the medial umbilical ligament was carried out as follows: A 2 mm skin incision was made at the body surface projection 1–2 cm lateral to the affected internal inguinal ring, and a non-absorbable suture was preinstalled on the fascial closure device. Under laparoscopic guidance, the fascial closure device vertically punctured the abdominal wall and peritoneum through the incision to access the abdominal cavity, then pierced the medial umbilical ligament from outside to inside at its edge and released one end of the suture medial to the ligament. The fascial closure device retreated extraperitoneally along the original route, retaining one suture end inside the abdomen and the other outside the body. The fascial closure device was punctured again through the same incision, with the peritoneal entry site several millimeters apart from the first puncture. The jaws captured the intraperitoneal suture and pulled it out through the abdominal wall. The suture was tightened externally, and laparoscopic observation confirmed that the medial umbilical ligament was fixed to the abdominal wall and covered Hesselbach's triangle.

**Figure 5:**
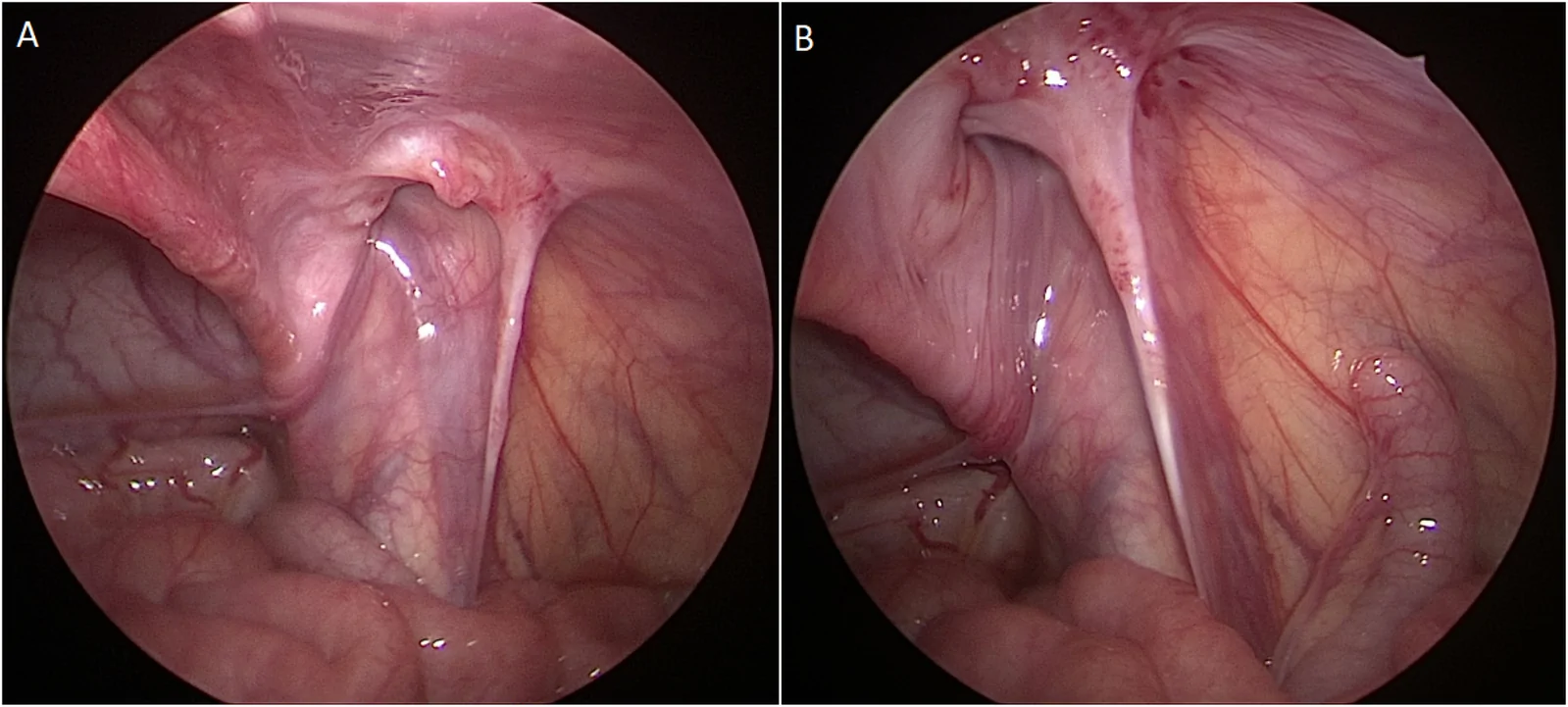
Patient 5. **(A)** Right direct inguinal hernia. **(B)** After repair.

**Figure 6:**
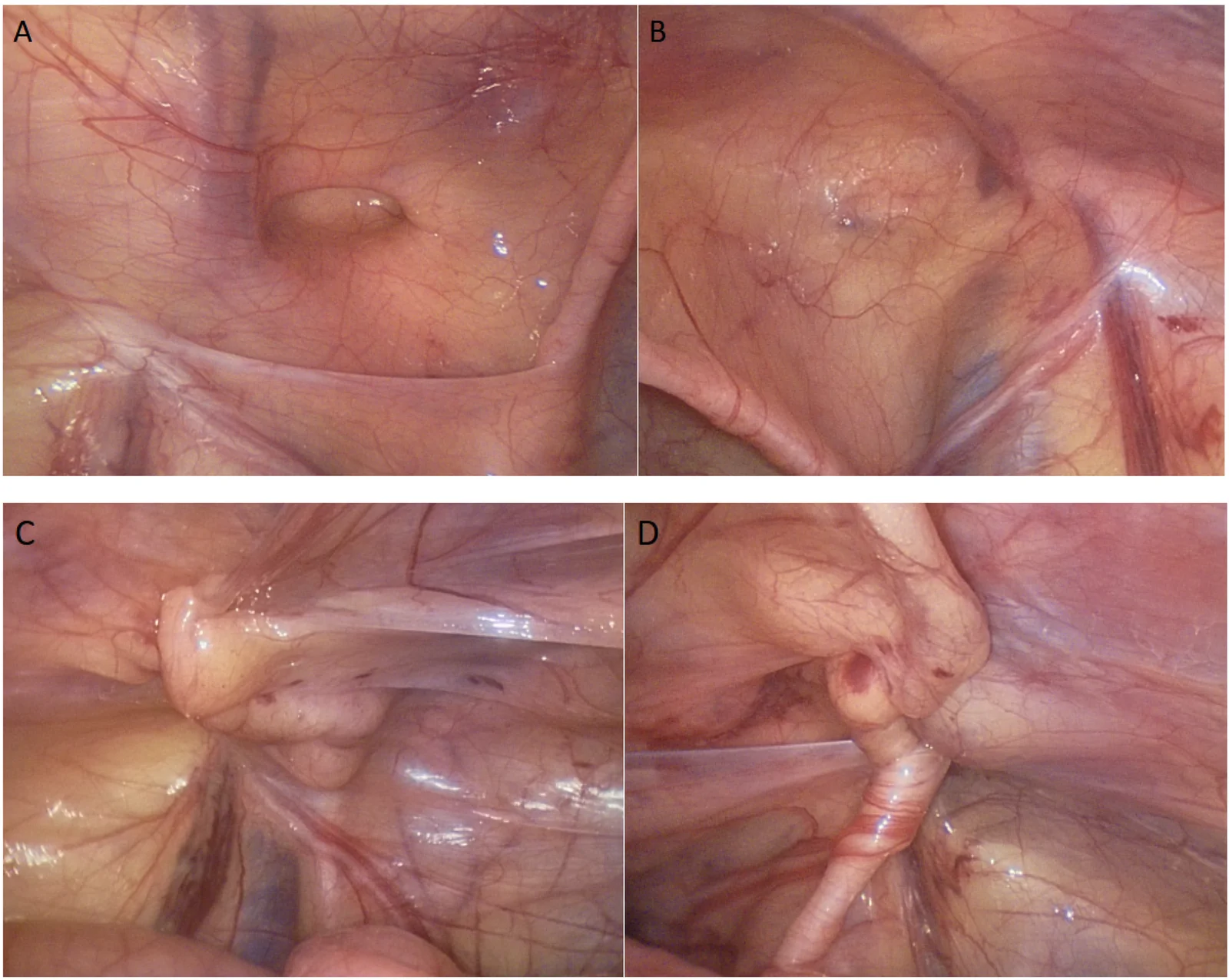
Patient 6. **(A)** Left direct inguinal hernia. **(B)** Right direct inguinal hernia. **(C)** Left direct inguinal hernia after repair. **(D)** Right direct inguinal hernia after repair.

Patient 6 had bilateral direct inguinal hernia (Figures 6A,B) and received combined laparoscopic ligation of direct inguinal hernia sac and laparoscopic medial umbilical ligament reinforcement. The specific operative procedure combined the surgical steps performed on Patient 3 and Patient 4. Laparoscopic examination confirmed that the medial umbilical ligament was anchored to the abdominal wall and covered Hesselbach's triangle (Figures 6C,D).

### Outcome definitions

The primary outcome of this retrospective case series was hernia recurrence during long-term postoperative follow-up. Recurrence was defined as re-emergence of visceral protrusion through the weakened Hesselbach's triangle confirmed by laparoscopic exploration or ultrasonography after initial repair, with follow-up ranging from 5 to 82 months until June 2026. Recurrence status was used as the core endpoint to compare the long-term efficacy of four laparoscopic repair techniques for pediatric direct inguinal hernia.

Predefined secondary outcomes included three categories of indicators:

1. Intraoperative safety parameters: intraoperative injury to vas deferens, spermatic vessels, viscera, bladder or inguinal nerves, intraoperative blood loss, and total operative time;
2. Short-term postoperative recovery and early complications: time to oral liquid intake, time to resume daily activities, and short-term adverse events including incision infection and subcutaneous hematoma;
3. Long-term adverse events other than recurrence: testicular atrophy and chronic inguinal pain.

All outcome data were extracted uniformly from electronic medical records, operative notes, and outpatient follow-up archives prior to statistical summary, without *post-hoc* adjustment of primary or secondary endpoints to avoid selective reporting bias.

## Results

A total of six male pediatric patients met the inclusion criteria, with none meeting the exclusion criteria. Their ages ranged from 2 years and 1 month to 7 years and 3 months, with a median age of 3 years and 9 months. All children were tentatively diagnosed with indirect inguinal hernia by preoperative physical examination and underwent laparoscopic surgical repair. All operations were performed by the same surgical team. Intraoperatively, the necks of all hernia sacs were located medial to the inferior epigastric vessels, and combined indirect inguinal hernia was excluded; a definitive diagnosis of direct inguinal hernia was therefore established. There were 2 cases of left direct inguinal hernia, 3 cases of right direct inguinal hernia, and 1 case of bilateral direct inguinal hernia. The operation duration ranged from 5 to 35 min, with a median of 7 min. All operations were completed smoothly with no obvious bleeding during intraperitoneal manipulation. No intraoperative complications such as injuries to the vas deferens, spermatic vessels, testis, intestine, bladder, external iliac vessels or peripheral nerve tissues were observed. Standardized symptomatic care was provided to all patients postoperatively. Liquid diet was initiated 6–8 h after surgery, and patients were encouraged to get out of bed and resume routine activities 24 h postoperatively. No short-term complications including incision infection or hematoma were detected during early postoperative follow-up, and all children recovered uneventfully and were discharged. All patients completed postoperative follow-up, with the last follow-up conducted in June 2026 and a follow-up interval ranging from 5 to 82 months. Long-term follow-up demonstrated no long-term complications including hernia recurrence, testicular atrophy and chronic inguinal pain in all patients, with favorable cosmetic results obtained (Table 1). No formal statistical comparison was performed because of the extremely limited sample size.

## Discussion

**Figure 7:**
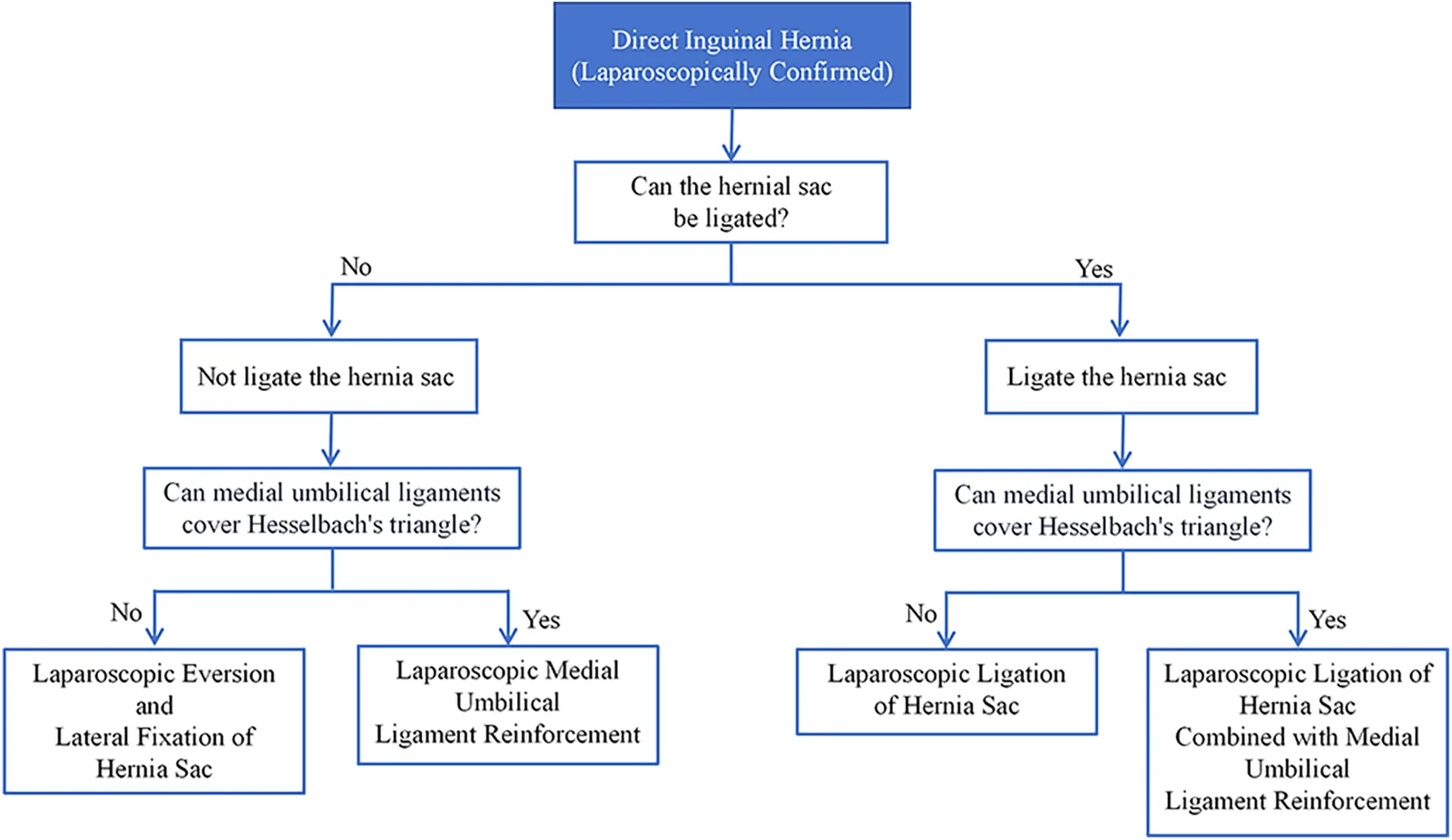
Laparoscopic treatment strategy for pediatric direct inguinal hernia.

Patients 1 and 2 underwent laparoscopic eversion and lateral fixation of direct inguinal hernia sac, a novel surgical technique that has not been previously reported. The indications for adopting this procedure are as follows. First, the tissues around the hernia sac neck were highly mobile in these two patients, making effective and reliable percutaneous ligation of the direct hernia sac unachievable with the fascial closure device. Second, the medial umbilical ligaments of the patients (especially Patient 1) were short and slender, failing to cover Hesselbach's triangle and anchor lateral to the internal inguinal ring. Third, the hernia sac tissues of the two children were thick and tough. Therefore, everting the base of the direct hernia sac and traction-fixing it lateral to the internal inguinal ring represents a more minimally invasive approach that rationally utilizes the fascial closure device without establishing an additional working port. Essentially, this technique also achieves coverage of the direct hernia triangle by the hernia sac tissue. The last follow-up durations for the two patients reached 4 years and 4 months, and 6 years and 9 months respectively, with no recurrence of direct inguinal hernia observed. This technique is suitable for patients with direct inguinal hernia who meet the above three conditions. The application of a fascial closure device is more minimally invasive and shortens operative time compared with placing an additional working trocar; however, fascial closure devices are mostly disposable consumables, which will incur extra medical costs. An alternative surgical option for such hernias involves creating an extra working port to perform laparoscopic peritoneal incision and repair (2, 4, 5). Patients 3 and 5 received laparoscopic high ligation of direct inguinal hernia sac. Satisfactory closure of the hernia sac was accomplished via simple ligation without additional medial umbilical ligament reinforcement. Generally, the distal segment of the hernia sac in pediatric direct inguinal hernia is shallower than that of indirect inguinal hernia. Consequently, during laparoscopic percutaneous ligation of direct hernia sac, the gliding movement of the fascial closure device at the hernia sac base results in less secure ligation at the sac neck compared with indirect hernia repair. Nevertheless, during the operations for Patients 3 and 5, the extraperitoneal dissection plane of the fascial closure device was not merely attached to the peritoneum, but also incorporated partial iliopubic tract and transversalis fascia into the suture loop. We speculate that this anatomical incorporation accounted for the absence of hernia recurrence during follow-up in these two patients. However, the suturing of iliopubic tract and transversalis fascia cannot be visualized under direct laparoscopy during high ligation of direct hernia sac, which leads to uncontrollable intraoperative quality of this procedure. Patient 4 presented with a 1-month history of reducible inguinal mass on the right side. Intraoperative exploration confirmed complete closure of the internal inguinal ring, excluded concurrent indirect inguinal hernia, and verified the diagnosis of direct inguinal hernia. Hernia sac ligation was not performed due to an indistinct hernia sac neck, and laparoscopic medial umbilical ligament reinforcement and fixation was conducted instead. Patient 6 suffered from bilateral direct inguinal hernia and was treated with combined laparoscopic percutaneous high ligation of direct inguinal hernia sac and medial umbilical ligament reinforcement suture. This combined procedure is the most widely reported laparoscopic technique for pediatric direct inguinal hernia and requires the use of a fascial closure device. The decision flowchart of the four surgical approaches is shown in Figure 7.

The sequential selection of the four distinct laparoscopic repair techniques administered to the six enrolled patients reflects the gradual accumulation of our institutional surgical experience in managing pediatric direct inguinal hernias, rather than random intraoperative assignment of surgical procedures. The first two patients (Patient 1 and Patient 2) were treated with the novel technique of laparoscopic hernia sac eversion and lateral fixation. This approach was adopted for scenarios where the hernia sac neck exhibited excessive mobility precluding safe circumferential ligation, allowing full utilization of autologous tissue to obliterate the hernia sac. We performed isolated high ligation of the hernia sac for Patients 3 and 5 after intraoperatively confirming that secure ligation of the hernia sac could be reliably achieved. For Patient 4, the indistinct, ill-defined anatomical boundary of the hernia sac neck made complete sac ligation technically unfeasible; we therefore switched to standalone medial umbilical ligament reinforcement as a salvage monotherapy. Combined high ligation of the hernia sac plus medial umbilical ligament reinforcement targets the two core pathological defects underlying pediatric direct inguinal hernia simultaneously: it fully obliterates the peritoneal hernia orifice via sac ligation, and delivers durable reinforcement to the congenitally weak Hesselbach's triangle using tension-free autologous medial umbilical ligament tissue. This combined technique was not arbitrarily selected solely for the sixth patient; instead, it represents an optimized strategy refined through iterative technical adjustments and intraoperative anatomical observations gathered from the preceding five cases.

Tao et al. conducted a retrospective study enrolling 10 children with intraoperatively confirmed direct inguinal hernia, all managed with laparoscopic ligation of hernia orifice plus reinforcement repair using the medial margin of medial umbilical ligament. No hernia recurrence was documented after 12 months of follow-up. The medial umbilical ligament in children is well-developed, tough, and possesses favorable tension and elasticity. As autologous tissue, it avoids the risks of foreign body rejection and infection associated with synthetic meshes, and exerts no adverse impacts on the development of the abdominal wall and reproductive system. The majority of children present with cord-like medial umbilical ligaments that suffice for intraoperative coverage and reinforcement, effectively strengthening Hesselbach's triangle and reducing recurrence rates (6). Bahadir et al. retrospectively reviewed 7 pediatric patients with direct inguinal hernia. Four cases received laparoscopic percutaneous ligation of hernia orifice combined with medial umbilical ligament reinforcement, while three cases were converted to open surgery. Early in their clinical practice, several patients required conversion to open repair. After accumulating surgical experience, the authors uniformly adopted the modified percutaneous internal ring suturing (PIRS) technique for direct hernia, establishing a standardized minimally invasive protocol. Modified PIRS (percutaneous suturing with supplementary medial umbilical ligament reinforcement when necessary) is safe and feasible and can serve as the mainstream minimally invasive strategy (7). If a fascial closure device is unavailable, an extra working port must be created to complete laparoscopic suturing and repair. In 2000, F. Schier reported five children with direct inguinal hernia treated laparoscopically by suturing the hernia defect with 4-0 non-absorbable suture combined with medial umbilical ligament reinforcement (8). Schier proposed that laparoscopic repair of pediatric direct inguinal hernia is more challenging than that of indirect hernia, owing to the lack of robust and stable anatomical structures surrounding the defect, which results in insufficient tensile strength after simple suturing. Incorporating the adjacent medial umbilical ligament into the final suture enables autologous tissue coverage of the defect, enhances repair durability, and improves surgical reliability. All the above authors adopted laparoscopic ligation of hernia sac combined with medial umbilical ligament reinforcement. This surgical technique is also the most widely reported and commonly used procedure at present. On the basis of hernial sac ligation, autologous tissue is applied for augmented coverage. This approach features simple operation and a low recurrence rate.

Based on surgical experience from 10 pediatric patients with direct inguinal hernia, in 2004 F. Schier and J. Klizaite concluded that the anatomical basis of direct inguinal hernia lies in weakness of the posterior inguinal wall, with pathological features distinct from ordinary indirect hernia. The simplified suturing protocol for indirect hernia cannot be directly replicated. The peritoneum over the direct hernia region must be incised to fully expose deep anatomical structures of the posterior inguinal wall, and multiple sutures are required to close the defect and reinforce repair strength to lower recurrence risk (9). Zhang et al. described the emergency management of a 15-month-old infant with incarcerated left direct inguinal hernia. Laparoscopic fascial defect repair combined with medial umbilical ligament reinforcement was performed without synthetic mesh, consistent with the developmental characteristics of the pediatric abdominal wall. No recurrence was observed at the 6-month follow-up (10). Li et al. reported three male children with direct inguinal hernia with a mean age of 36 months (range, 24–48 months). The direct hernia defects were closed with 2-0 polypropylene suture and further covered and reinforced by the medial umbilical ligament. No hernia recurrence was detected after 12–48 months of follow-up (11). Both Zhang et al. and Li et al. completed defect repair without peritoneal incision. Some scholars advocate resecting the hernia sac or incising it to perform extraperitoneal transversalis fascia repair. Sang Kyung Lee treated 10 children with direct inguinal hernia and 11 patients with metachronous contralateral indirect inguinal hernia via laparoscopic iliopubic tract repair, in which non-absorbable sutures were used to fix the iliopubic tract to the transversalis fascia to reinforce the posterior inguinal wall. Long-term follow-up revealed zero recurrence (2). Wafa et al. retrospectively analyzed nine children with direct inguinal hernia managed with mesh-free laparoscopic transabdominal preperitoneal repair. Only one case exhibited tension at the repair site and received auxiliary medial umbilical ligament reinforcement. The median follow-up duration was 24 months (range, 2 months to 5 years), and no hernia recurrence occurred in all subjects. The authors stated that the extraperitoneal approach allows complete visualization of fasciae, muscular arches, and inguinal ligaments, enabling accurate identification of defect margins and thorough anatomical repair (5). Esposito et al. summarized surgical experience of seven children with direct inguinal hernia, all presenting with defects exceeding 10 mm in diameter and fatty pads attached to the hernia sac. Complete excision of fatty pads via hook electrocoagulation was identified as a key step to prevent recurrence. The fascial and peritoneal defects were closed with interrupted non-absorbable sutures, and the medial umbilical ligament was utilized as an autologous tissue patch to cover and reinforce the defect for tension-free repair (4). All the aforementioned authors performed laparoscopic resection of the hernial sac. In some cases, extraperitoneal transversalis fascia repair was combined to reinforce the posterior inguinal wall, while medial umbilical ligament coverage and reinforcement was adopted in other cases. This surgical approach requires two trocars. Its operative procedures are more complicated than those of laparoscopic ligation of hernia sac combined with medial umbilical ligament reinforcement, with one additional trocar needed.

Bandi et al. treated a 3-year-old boy with complete resection of the hernia sac and peritoneal closure using 3-0 polypropylene suture without additional posterior inguinal wall reinforcement. The patient was discharged on the postoperative day 1 and remained recurrence-free after 1 year of follow-up (12). Sung Ryul Lee demonstrated that recurrent hernia after pediatric indirect inguinal hernia repair arises from weakness of inguinal muscles and fasciae, an etiology shared with pediatric direct inguinal hernia. Laparoscopic suturing of iliopubic tract to transversalis fascia for posterior inguinal wall reinforcement effectively reduces re-recurrence rates in children with recurrent indirect inguinal hernia (13). For direct hernias recurring after the abovementioned autologous tissue repairs, tension-free mesh repair has been reported as an alternative. Chin et al. reported a critically ill extremely preterm infant born at 25 + 5 weeks gestation with initial bilateral indirect inguinal hernia that recurred after laparoscopic repair and progressed to bilateral saddle hernia. Reoperation failed to achieve radical cure, resulting in left indirect and right direct inguinal hernia (14). Multiple autologous tissue repairs including internal ring suturing and medial umbilical ligament reinforcement were ineffective. Open Lichtenstein tension-free mesh repair was ultimately performed for the left recurrent direct hernia when the child reached 3 years old, with no hernia recurrence or related complications noted at the 12-month follow-up. This case indicates that autologous tissue repair alone yields suboptimal long-term outcomes. For children with recurrent direct inguinal hernia after multiple failed conventional autologous tissue procedures, synthetic mesh reinforcement may be cautiously adopted after strict indication evaluation (14). Reports on surgical techniques for direct inguinal hernia in older children are scarce. Bandi et al. performed open Desarda tension-free hernia repair on a 15-year-old adolescent with direct inguinal hernia (12). All the above reports were individual case studies. These surgical methods have been sparsely documented, resulting in limited reference value.

### Limitations

This study has several limitations. First, it is a single-center retrospective case series with an extremely small sample size of only six male patients, which limits the generalizability of the findings to female patients or broader pediatric populations. Second, as a retrospective study, inherent selection bias and information bias cannot be fully excluded, and we were unable to control for intraoperative decision-making regarding surgical technique selection. Third, follow-up duration varied widely across cases (5 to 82 months); notably, the patient treated with the combined technique had only a 5-month follow-up, which limits conclusions regarding long-term recurrence for this specific approach, and the inconsistent follow-up period may affect the comparability of long-term recurrence outcomes. Fourth, no standardized patient-reported outcome measures were collected due to the retrospective design. Finally, the absence of a control group prevents definitive comparative efficacy conclusions among the four techniques.

### Clinical implications and future directions

Based on our preliminary experience, we propose a clinical decision-making framework for pediatric direct inguinal hernia: combined hernia sac ligation with medial umbilical ligament reinforcement is preferred as the first-line option when anatomical conditions permit; for patients with indistinct hernia sac necks, isolated medial umbilical ligament reinforcement is a feasible alternative; and the novel eversion-lateral fixation technique can be considered for cases with mobile hernia sac necks and underdeveloped medial umbilical ligaments.

Future multicenter, large-sample prospective cohort studies are warranted to validate the long-term efficacy and safety of these techniques, and randomized controlled trials are needed to directly compare recurrence rates among different repair strategies.

## Conclusion

Laparoscopic surgery delivers prominent strengths including high safety, reliable efficacy and minimal trauma for pediatric direct inguinal hernia repair. It provides a clear intraoperative visual field, enabling precise exploration of inguinal anatomical structures and effective correction of preoperative misdiagnosis. Theoretically, isolated hernia sac ligation can achieve basic repair of pediatric direct inguinal hernia; however, it carries drawbacks such as insecure ligation and suture displacement, which may confer a relatively higher long-term risk of hernia recurrence. Hernia sac ligation combined with medial umbilical ligament reinforcement may strengthen tissue integrity at the direct hernia repair site in theory and compensate for congenital anatomical weakness of the transversalis fascia. Based on our limited single-center experience, the specific surgical technique should be selected according to whether the hernia sac can be easily ligated and whether the medial umbilical ligament is sufficient to cover Hesselbach's triangle, and larger cohort studies are needed to confirm the clinical performance of each repair strategy.

## Data Availability

The original contributions presented in the study are included in the article/Supplementary Material, further inquiries can be directed to the corresponding author/s.

## References

[1] Gödeke J Muensterer OJ. Femoral, direct, and rare inguinal hernias in children-an update. Eur J Pediatr Surg. (2017) 27(6):484–94. 10.1055/s-0037-1608684 29141259

[2] Lee SR Lee SK. Laparoscopic iliopubic tract repair for acquired pediatric inguinal hernia. J Laparoendosc Adv Surg Tech A. (2026) 36(7):537–42. 10.1177/10926429251406486 41661108

[3] Mathew G Sohrabi C Franchi T Nicola M Kerwan A Agha R, PROCESS Group. Preferred reporting of case series in surgery (PROCESS) 2023 guidelines. Int J Surg. (2023) 109(12):3760–9. 10.1097/JS9.0000000000000940 37988417 PMC10720832

[4] Esposito C Alicchio F Giurin I Castellano M Settimi A. Technical standardization of laparoscopic direct hernia repair in pediatric patients. J Laparoendosc Adv Surg Tech A. (2012) 22(1):113–6. 10.1089/lap.2011.0324 22044564

[5] Wafa TA El-Saied AW Darwish M Elshafey A Elayoty M Elbatarny A. Trans-abdominal pre-peritoneal (TAPP) approach for anatomical repair of direct inguinal hernia in children: could it be the new standardized approach? J Pediatr Surg. (2024) 59(12):161682. 10.1016/j.jpedsurg.2024.08.022 39242219

[6] Chengpin T Yongsheng C Changkun M. Evaluation and analysis of the clinical effects of laparoscopic surgery for pediatric direct inguinal hernia. J Laparoendosc Adv Surg Tech A. (2025) 35(1):89–93. 10.1089/lap.2024.0158 39501870

[7] Bahadir K Gerus S Sozduyar S Ates U Gollu G Kologlu M. Rare findings of indirect inguinal hernia repair during laparoscopic PIRS technique in children: a two-center audit. Front Pediatr. (2026) 14:1742114. 10.3389/fped.2026.1742114 41809522 PMC12968235

[8] Schier F. Direct inguinal hernias in children: laparoscopic aspects. Pediatr Surg Int. (2000) 16(8):562–4. 10.1007/s003830000431 11149394

[9] Schier F Klizaite J. Rare inguinal hernia forms in children. Pediatr Surg Int. (2004) 20(10):748–52. 10.1007/s00383-004-1291-7 15503064

[10] Zhang G Sun M Shu J Bian H Guo Q Yang J. Laparoscopic closure of the fascial defect combined with medial umbilical ligament reinforcement for incarcerated pediatric direct inguinal hernia: a case report with emergency management insights. Front Med. (2026) 13:1840599. 10.3389/fmed.2026.1840599 PMC13171553 42145714

[11] Li M Li F Peng Q. Laparoscopic repair of direct inguinal hernia in children. Asian J Surg. (2024) 47(12):5379–80. 10.1016/j.asjsur.2024.06.063 38951054

[12] Bandi S Singh S Prakash D. A new perspective on direct inguinal hernia repair in children: a unique technique for enhanced outcomes. J Indian Assoc Pediatr Surg. (2025) 30(5):674–6. 10.4103/jiaps.jiaps_80_25 40950613 PMC12425378

[13] Lee SR. Laparoscopic iliopubic tract repair to treat recurrent pediatric inguinal hernia. Surg Endosc. (2022) 36(6):4321–7. 10.1007/s00464-021-08776-5 34694490 PMC9085696

[14] Chin SE Yen JB Wang SH. Laparoscopic identification and management of a pediatric direct inguinal hernia in a high-risk infant: a case report. Urol Case Rep. (2026) 66:103403. 10.1016/j.eucr.2026.103403 41884855 PMC13011051

